# Transmembrane Protein 39A Promotes the Replication of Encephalomyocarditis Virus *via* Autophagy Pathway

**DOI:** 10.3389/fmicb.2019.02680

**Published:** 2019-11-29

**Authors:** Xiangrong Li, Ruixian Ma, Qian Li, Shengjun Li, Haixia Zhang, Jingying Xie, Jialin Bai, Adi Idris, Ruofei Feng

**Affiliations:** ^1^Key Laboratory of Biotechnology and Bioengineering of State Ethnic Affairs Commission, Biomedical Research Center, Northwest Minzu University, Lanzhou, China; ^2^Gansu Tech Innovation Center of Animal Cell, Biomedical Research Center, Lanzhou, China; ^3^Life Science and Engineering College, Northwest Minzu University, Lanzhou, China; ^4^College of Veterinary Medicine, Gansu Agricultural University, Lanzhou, China; ^5^School of Medical Science, Menzies Health Institute Queensland, Griffith University, Gold Coast, QLD, Australia

**Keywords:** transmembrane protein 39A, encephalomyocarditis virus, replication, autophagy, ATG7

## Abstract

Encephalomyocarditis virus (EMCV) causes encephalitis, myocarditis, neuropathy, reproductive disorders, and diabetes in animals. EMCV is known to induce cell autophagy; however, the molecular mechanisms underlying this remain unclear. Here, we show that the type III-transmembrane protein, transmembrane protein 39A (TMEM39A), plays a critical role in EMCV replication. We showed that EMCV GS01 strain infection upregulated TMEM39A expression. Importantly, EMCV induced autophagy in a range of host cells. The autophagy chemical inhibitor, 3-MA, inhibited EMCV replication and reduced TMEM39A expression. This is the first study demonstrating TMEM39A promoting the replication of EMCV *via* autophagy. Overall, we show that TMEM39A plays a positive regulatory role in EMCV proliferation and that TMEM39A expression is dependent on the autophagy pathway.

## Introduction

Encephalomyocarditis virus (EMCV) is a positive sense single-stranded RNA virus belonging to the *Picornaviridae* family (Koenen, 2006). EMCV is commonly used to study innate immune responses toward double-stranded RNA (dsRNA) (Carocci and Bakkali, 2012). EMCV causes encephalitis, myocarditis, neuropathy, reproductive disorders, and diabetes in domestic animals, rodents, and primates (Carocci and Bakkali, 2012). EMCV infection is common in large-scale pig farms in China (Zhang et al., 2017). EMCV can also infect humans as the serum prevalence rate of EMCV in healthy Chinese people is approximately 30.56% (Feng et al., 2015). Therefore, an in-depth understanding of EMCV has important implications for public health (Oberste et al., 2009). EMCV life cycle and molecular epidemiology are well studied (Bai et al., 2014; Feng et al., 2015, 2015; Liu et al., 2016; Luo et al., 2017; Zhang et al., 2017). However, little is known about the factors that influence EMCV replication. In a yeast two-hybrid screening, we previously found that transmembrane protein 39A (TMEM39A) interacted with EMCV capsid proteins, VP1 and VP2. TMEM39A belongs to the type III-transmembrane protein family and has eight transmembrane domains (Tran et al., 2017). TMEM39A is known to be associated with autoimmune diseases, such as systemic lupus erythematosus and multiple sclerosis (Mccauley et al., 2010; Lessard et al., 2012; Varade et al., 2012; Sheng et al., 2015; You et al., 2015; Wagner et al., 2017). Furthermore, TMEM39A has been proposed to be a novel marker for the diagnosis of glioma and other tumors (Park et al., 2017).

Previous studies have shown that EMCV infection can induce autophagy in host cells (Zhang et al., 2011); however, the underlying molecular mechanism of EMCV-induced autophagy remains elusive. Cell autophagy (or autophagocytosis) is the phenomenon of “self-eating” within eukaryotic cells, which is a ubiquitous mechanism that refers to the use of lysosomes to degrade the damaged organelles and macromolecular materials, a process that is under the regulation of autophagy-related genes (Atg) (Levine, 2005; Levine and Deretic, 2007; Schmid and Münz, 2007). The conversion of microtubule-associated protein 1 light chain 3 (MAP1LC3/LC3) and the degradation of sequestosome 1 (SQSTM1, p62) are considered the primary indicators of autophagy (Xiao et al., 2016). LC3 is first cleaved by ATG4B to form LC3-I, which is subsequently lipidated by phosphatidylethanolamine (PE) to form LC3-II *via* an interaction with ATG3 and ATG7 (You et al., 2019). In this study, we show that TMEM39A directly interacts with EMCV VP1 and VP2 and played a positive regulatory role in the proliferation of EMCV. We show that EMCV induced complete autophagy in a number of cell lines. Overexpression of TMEM39A upregulated LC3B-II and ATG7 and downregulated SQSTM1 expression. Consequently, ATG7 and LC3B expressions were decreased when TMEM39A was knocked down. Moreover, we showed that the expression of the EMCV capsid protein, VP2, increased the expression of TMEM39A and ATG7 and that the autophagy inhibitor, 3-MA, inhibited the replication of EMCV and the expression of TMEM39A. Overall, these results verify a novel role of TMEM39A in positively regulating the replication of EMCV *via* autophagy-dependent pathway. Our findings provide novel ideas for clarifying the role of TMEM39A in viral infections.

## Materials and Methods

### Cells, Virus, and Plasmids

C2C12, BHK-21, and HEK293 cells were obtained from ATCC and cultured in Dulbecco’s modified Eagle’s medium (DMEM; Lanzhou Minhai Bio-engineering) supplemented with 10% (v/v) newborn bovine serum (NBS; Lanzhou Minhai Bio-engineering) in a 37°C incubator. We used the EMCV GS01 strain in this study and was isolated as previously described (Feng et al., 2015). pET28a, pET30a, His-VP1, His-VP2, His-VP3, pCMV-HA, HA-VP1, HA-VP2, pGEX-6P-1, GST-TMEM39A, pCMV-Myc, Myc-EGFP, Myc-TMEM39A, pcDNA3.1(+), 3.1-TMEM39A, pDsRed-monomer-N1, Red-LC3, pCMV6-Entry, and Entry-TMEM39A were all cloned and produced in-house in our laboratory.

### Antibodies and Reagents

Anti-HA antibody (A02040) was purchased from Abbkine. Antibodies against ACTB (ab6276), 6 × His tag (ab18184), GST (ab92), and TMEM39A (ab175618) were purchased from Abcam. Anti-LC3B antibody (14600-1-AP) was purchased from Proteintech. Anti-ATG7 antibody (AA820) was purchased from Beyotime. Anti-SQSTM1/p62 antibody (WH098631) was purchased from Abclonal. Peroxidase AffiniPure goat anti-rabbit IgG (H + L) (111-035-003) and anti-mouse IgG (H + L) (115-035-003) were purchased from Jackson ImmunoResearch Laboratories. RIPA (P0013K), NP40 (P0013F) and PMSF (ST506-2) were purchased from Beyotime. Lipofectamine™ 2000 (11668019), Pierce™ GST protein interaction pull-down kit (21516), and protein G dynabeads™ (10004D) were purchased from ThermoFisher. 3-(4,5-Dimethylthiazol-2-yl)-2,5-diphenyltetrazolium bromide (MTT, M5655) and 3-methyladenine (3-MA, M9281) were purchased from Sigma-Aldrich.

### Quantitative Reverse Transcription Real-Time Polymerase Chain Reaction

Total RNA was extracted from cells and supernatant using the RNAiso Plus kit (Takara, 9109) according to the manufacturer’s protocol, and RNA was reverse transcribed. RT-qPCR was done using the PowerUp™ SYBR Green Master Mix (ThermoFisher, A25742) or the Premix Ex Taq Probe qPCR (Takara, RR390A). The expression level of each target gene was calculated by normalization to ACTB using the ΔΔCT method, and the ratio of the two genes in the control group was normalized to 1. All RT-qPCR primer sequences are listed in Table 1.

**Table 1:** Sequences of the RT-qPCR primers.

Name	Primer Sequence
Homo-*TMEM39A*-qF	5′-GGACCCTCGTCAATCTCTTTC-3′
Homo-*TMEM39A*-qR Homo-*ACTB*-qF	5′-TACTGCCTCGTGCTGAACC-3′ 5′-TGGCACCCAGCACAATGAA-3′
Homo-*ACTB*-qR Homo-*LC3B*-qF Homo-*LC3B*-qR Mus-*ATG7*-qF Mus-*ATG7*-qR Mus-*ATG3*-qF Mus-*ATG3*-qR Mus-*ATF4*-qF Mus-*ATF4*-qR Mus-*ACTB*-qF Mus-*ACTB*-qR Mus-*TMEM39A*-qF2 Mus-*TMEM39A*-qR2 EMCV-*3D*-qF EMCV-*3D*-qR EMCV-probe	5′-CTAAGTCATAGTCCGCCTAGAAGCA-3′ 5′-GAGAAGCAGCTTCCTGTTCTGG-3′ 5′-GTGTCCGTTCACCAACAGGAAG-3′ 5′-CCTGTGAGCTTGGATCAAAGGC-3′ 5′-GAGCAAGGAGACCAGAACAGTG-3′ 5′-TAAGGCTGACGCTGGAGGTGAA-3′ 5′-GTGCTCAACTGTTAAAGGCTGCC-3′ 5′-AACCTCATGGGTTCTCCAGCGA-3′ 5′-CTCCAACATCCAATCTGTCCCG-3′ 5′-CATTGCTGACAGGATGCAGAAGG-3′ 5′-TGCTGGAAGGTGGACAGTGAGG-3′ 5′-CCTCAATCTCCTGTTCCTTGGC-3′ 5′-TCCTCTACTGCCTGGTGCTGAA-3′ 5′-GTCATACTATCGTCCAGGGACTCTAT-3′ 5′-CATCTGTACTCCACACTCTCGAATG-3′ 5′(FAM)CACTTCGATCACTATGCTTGCCGTT(Eclipse)-3′

### Immunoblotting

Cell monolayers were incubated on ice with RIPA lysis buffer (Beyotime, P0013K) containing PMSF (Beyotime, ST506-2). Proteins were separated on 15% SDS-PAGE gels and subsequently transferred to PVDF membranes (Merck, ISEQ00010) using a Trans-Blot Turbo™ RTA Mini PVDF Transfer Kit (Bio-rad, 1704272). The membranes were then sequentially incubated with primary antibodies and peroxidase affinipure goat anti-rabbit or mouse IgG (H + L). Specific protein bands were analyzed using an ECL kit (Bio-rad, 1705060).

### Co-immunoprecipitation and Pull-Down Assays

Protein G dynabeads™ (ThermoFisher, 10004D) were mixed with the corresponding antibody overnight at 4°C. For Co-IP assays, cells were incubated in NP40 lysis buffer (Beyotime, P0013F) containing PMSF (Beyotime, ST506-2). The following day cell lysates containing the antigen was added to the Protein G mixture. The bead-Ab-Ag complexes were washed five times by gentle pipetting and subjected to immunoblotting. For the pull-down assays, His-labeled VP1, VP2, and VP3, pGEX-6P-1, and GST-TMEM39A were expressed in *E.coli* BL21 strain. GST alone or GST-TMEM39A protein was mixed with the glutathione agarose resin (Thermo Fisher, 21516) and incubated overnight at 4°C. The following day, His-labeled proteins were added separately to the above mixture. After conjugation, the unbound proteins were washed away and subjected to immunoblotting.

### RNA Interference

Cells were seeded in six-well plates and allowed to reach 50% confluency. Cells were transfected with 150 nM specific siRNA using Lipofectamine™ 2000 (Invitrogen, 11668019) according to the manufacturer’s instructions. siRNAs targeting TMEM39A (GGACCCTCGTCAATCTCTT) and non-targeting control (NC) (ACGTGACACGTTCGGAGAA) were purchased from RiboBio. At 48 h post-transfection, knockdown efficiency of endogenous TMEM39A was confirmed by RT-qPCR and immunoblotting.

### Encephalomyocarditis Virus Infection and Titration of Viral Progeny

HEK293, C2C12 cells, and BHK-21 cells were infected with EMCV at a multiplicity of infection (MOI) of 0.0001 and cultured in DMEM/3%NBS for a further 24 h post-infection (hpi). Cells and another group of the mixture of cells and supernatants after repeated freezing and thawing for three times were harvested separately for RT-qPCR analysis and the TCID_50_ assay (Reed-Muench method).

### Cell Viability Assay

Cell viability was detected by the MTT assay as described previously (Zhang et al., 2011).

### Statistical Analysis

Results were displayed as the mean ± SD. Statistical analysis was performed using the Student’s *t*-test. *p* <0.05 was considered a statistically significant difference between two groups (^*^p < 0.05; ^**^p < 0.01; ^***^p < 0.001).

## Results

### Transmembrane Protein 39A Binds VP1 and VP2 Directly

**Figure 1:**
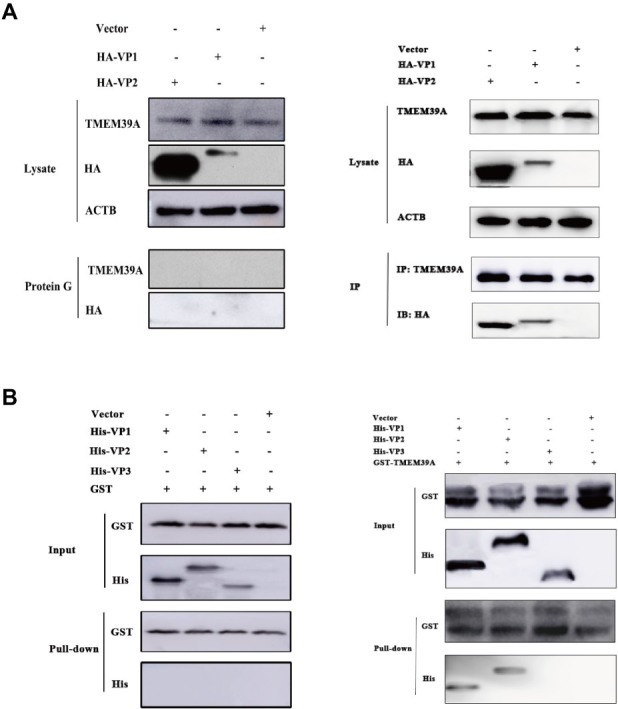
TMEM39A binds VP1 and VP2 *via* a direct interaction. **(A)** Endogenous TMEM39A interacted with HA-labeled VP1 and VP2 in a Co-IP assay. HEK293 cells were transfected with HA-VP1, HA-VP2, or empty vector for 48 h. **(B)** *In vitro* assay of the interaction between GST/GST-TMEM39A protein and His-VP1, His-VP2, or His-VP3.

We recently found that TMEM39A interacted with the EMCV capsid proteins, VP1 (BHK-21 cell cDNA library) and VP2 (HUVEC cell cDNA library) *via* the yeast two-hybrid assay. To verify this, we performed *in vitro* Co-IP and pull-down assays. Since HEK293 cells endogenously express TMEM39A, these cells were transfected with HA-labeled VP1 or VP2. As expected, overexpressed HA-labeled VP1 and VP2 co-precipitated with endogenous TMEM39A (Figure 1A). Similarly, GST-labeled TMEM39A pulled down His-labeled VP1 and VP2 but not VP3 (Figure 1B). These results suggest that TMEM39A directly interacts with EMCV VP1 and VP2 proteins.

### Transmembrane Protein 39A Plays a Positive Regulatory Role in the Replication of Encephalomyocarditis Virus

**Figure 2:**
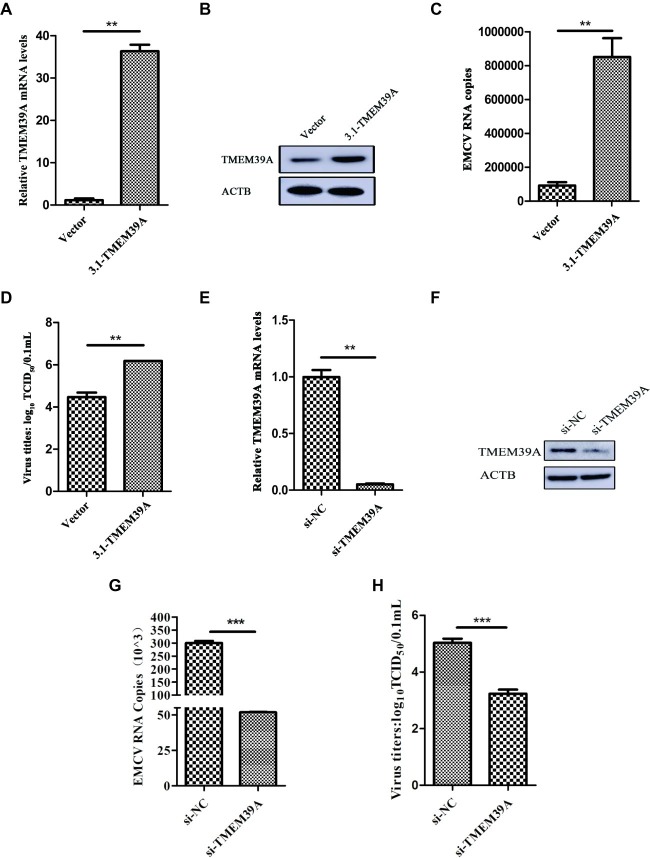
TMEM39A plays a positive regulatory role in the replication of EMCV. **(A)** RT-qPCR analysis of the mRNA expression level of TMEM39A in HEK293 cells transfected with 3.1-TMEM39A or pcDNA3.1(+) for 48 h. The level of TMEM39A mRNA was normalized to that of ACTB. **(B)** Immunoblotting of TMEM39A expression in HEK293 cells transfected with 3.1-TMEM39A or pcDNA3.1(+) for 48 h. ACTB was used as an internal reference. **(C)** HEK293 cells were transfection with 3.1-TMEM39A or pcDNA3.1(+) for 48 h and further infected with 0.0001 MOI EMCV GS01 strain at 24 hpi. Total RNA was extracted followed by RT-qPCR analysis of EMCV copies in the TMEM39A and empty vector groups. **(D)** TCID_50_ assay for viral titer detection in the mixture after repeated freezing and thawing of the 3.1-TMEM39A and pcDNA3.1(+) using the Reed-Muench method. **(E)** RT-qPCR analysis of the mRNA expression of TMEM39A in HEK293 cells transfected with 150 nM siRNA against TMEM39A or NC for 48 h. The level of TMEM39A mRNA was normalized to that of ACTB. **(F)** Immunoblotting of TMEM39A expression in HEK293 cells transfected with 150 nM siRNA against TMEM39A or NC for 48 h. ACTB was used as an internal reference. **(G)** HEK293 cells were transfection with 150 nM siRNA against TMEM39A or NC for 48 h and further infected with 0.0001 MOI EMCV GS01 strain at 24 hpi. Total RNA was extracted followed by RT-qPCR analysis of EMCV copies in the si-TMEM39A and si-NC groups. **(H)** TCID_50_ assay for viral titer detection in the mixture after repeated freezing and thawing of the si-TMEM39A and si-NC groups using the Reed-Muench method. Data are expressed as mean ± SD, *n* = 3. **p < 0.01, ***p < 0.001.

To further investigate the role of TMEM39A in EMCV infection, we overexpressed TMEM39A in HEK293 cells. TMEM39A mRNA and protein expression levels were significantly increased 48 h post-transfection (Figures 2A,B). Compared to the control, EMCV copy number and titer were markedly higher in the TMEM39A-transfected group (Figures 2C,D), indicating that TMEM39A overexpression increased EMCV replication. Furthermore, knockdown of TMEM39A (Figures 2E,F) suppressed the EMCV proliferation (Figures 2G,H). Taken together, these results indicate that TMEM39A played a positive regulatory role in EMCV replication.

### Encephalomyocarditis Virus GS01 Strain Infection Induces Autophagy in a Range of Different Host Cells

**Figure 3:**
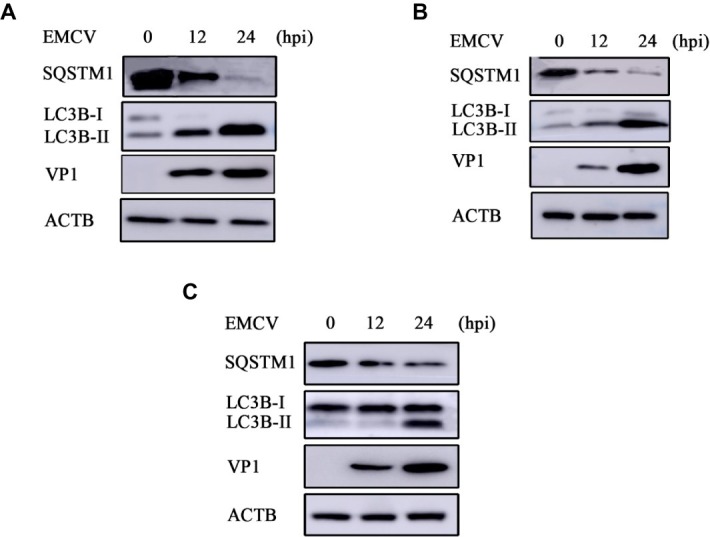
EMCV GS01 strain infection can induce complete autophagy. **(A)** Immunoblotting of the expression of VP1, LC3B, and SQSTM1 in BHK-21 cells which were infected with 0.0001 MOI EMCV GS01 strain at different times. ACTB was used as an internal reference. **(B)** Immunoblotting of the expression of VP1, LC3B, and SQSTM1 in C2C12 cells which were infected with 0.0001 MOI EMCV GS01 strain at different times. ACTB was used as an internal reference. **(C)** Immunoblotting of the expression of VP1, LC3B, and SQSTM1 in HEK293 cells which were infected with 0.0001 MOI EMCV GS01 strain at different times. ACTB was used as an internal reference.

Previous work has shown that EMCV HB10 infection can induce autophagy in BHK-21 cells and that autophagy promotes the replication of EMCV (Zhang et al., 2011). We wanted to see whether this could occur with the EMCV GS01 strain. We see a remarkable increase in LC3B-I to LC3B-II conversion in EMCV-infected BHK-21 cells at 12 and 24 hpi, and a decrease in SQSTM1 expression (Figure 3A), which is a marker of autophagy progression. Similar findings were observed in C2C12 cells (Figure 3B) and HEK293 cells (Figure 3C), although increase in LC3B-I to LC3B-II conversion was slightly delayed in HEK293 cells. Overall, we show that EMCV GS01 strain infection induced autophagy in different host cells.

### Transmembrane Protein 39A Promotes Encephalomyocarditis Virus Proliferation and Can Regulate the Autophagic Machinery

**Figure 4:**
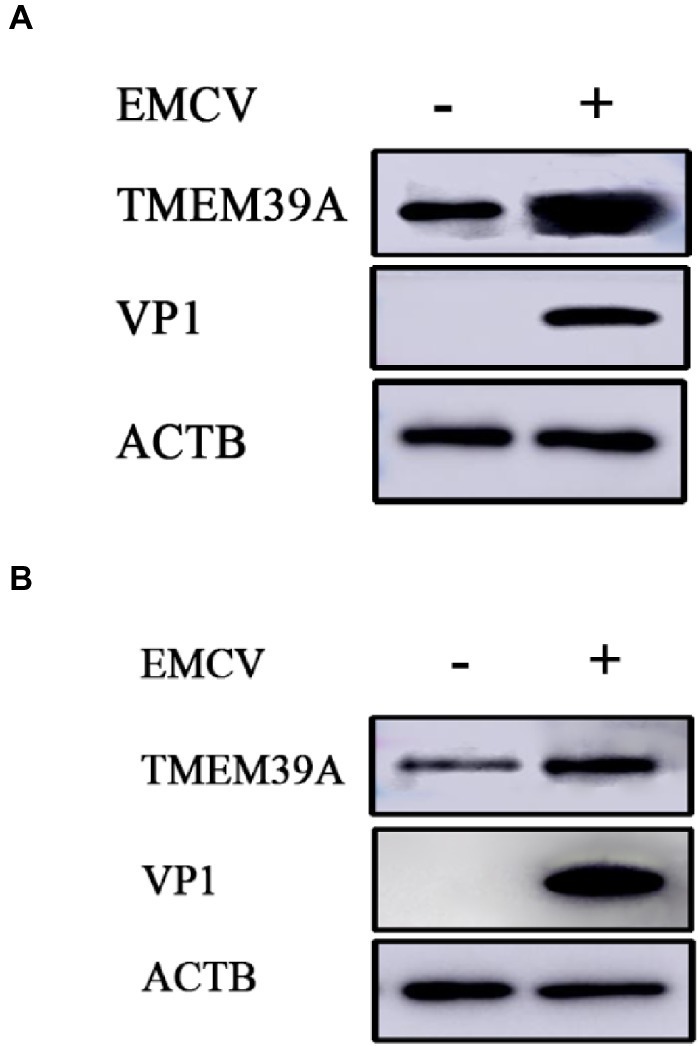
EMCV infection upregulates the expression of TMEM39A. **(A)** Immunoblotting of VP1 and TMEM39A expression in HEK293 cells which were infected with 0.0001 MOI EMCV GS01 strain at 24 hpi. ACTB was used as an internal reference. **(B)** Immunoblotting of VP1 and TMEM39A expression in C2C12 cells which were infected with 0.0001 MOI EMCV GS01 strain at 24 hpi. ACTB was used as an internal reference.

**Figure 5:**
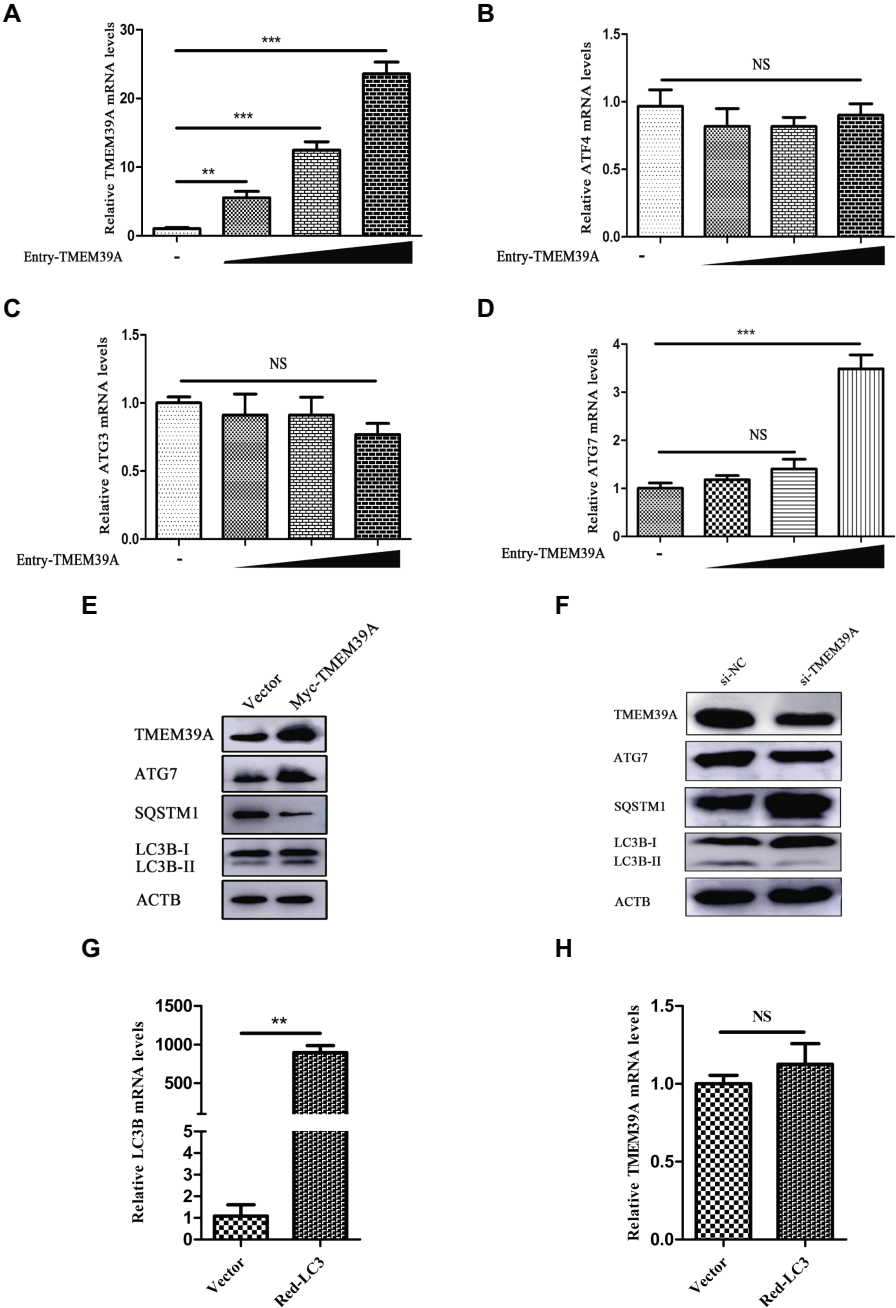
TMEM39A promotes the proliferation of EMCV *via* autophagy pathway. **(A)** RT-qPCR of the mRNA expression of TMEM39A in BHK-21 cells transfected with 0.5, 1, or 2 μg Entry-TMEM39A or 2 μg pCMV-Entry for 48 h. The mRNA level of TMEM39A was normalized to that of ACTB. **(B–D)** RT-qPCR of the mRNA expression of ATF4, ATG3 and ATG7 in BHK-21 cells transfected with 0.5, 1, or 2 μg Entry-TMEM39A or 2 μg pCMV-Entry for 48 h. The mRNA levels were normalized to that of ACTB. **(E)** Immunoblotting of TMEM39A, ATG7, LC3B, and SQSTM1 expression in HEK293 cells transfected with 2 μg Myc-TMEM39A or pCMV-Myc for 48 h. ACTB was used as an internal reference. **(F)** Immunoblotting of TMEM39A, ATG7, LC3B, and SQSTM1 expression in HEK293 cells transfected with siRNA against TMEM39A or NC for 48 h. ACTB was used as an internal reference. **(G)** RT-qPCR of the mRNA expression of LC3B in HEK293 cells transfected with 2 μg Red-LC3B or pDsRed-monomer-N1 for 48 h. The mRNA level of LC3B was normalized to that of ACTB. **(H)** RT-qPCR of the mRNA expression of TMEM39A in HEK293 cells transfected with 2 μg Red-LC3B or pDsRed-monomer-N1 for 48 h. The mRNA level of TMEM39A was normalized to that of ACTB. Data are expressed as mean ± SD, *n* = 3. **p < 0.01, ***p < 0.001.

**Figure 6:**
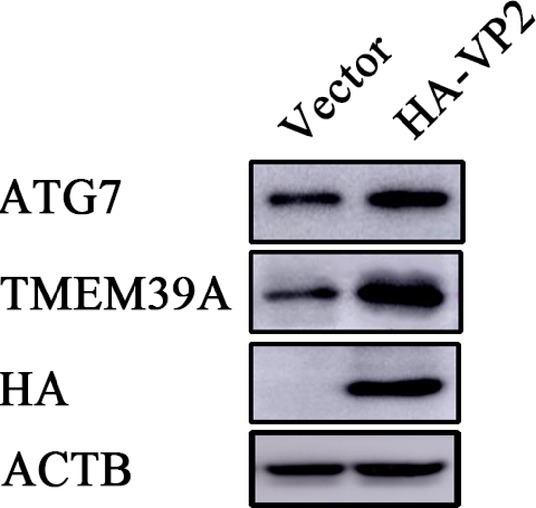
Overexpression of VP2 facilitates the expression of TMEM39A. Immunoblotting of HA, TMEM39A and ATG7 expression in HEK293 cells transfected with 2 μg HA-VP2 or pCMV-HA for 48 h. ACTB was used as an internal reference.

To investigate the effect of EMCV infection on TMEM39A expression, we evaluated the expression of TMEM39A in different cells by immunoblotting. We found that the TMEM39A was markedly upregulated in EMCV-infected HEK293 and C2C12 cells at 24 hpi (Figures 4A,B). Since the expression levels of TMEM39A were significantly upregulated and EMCV GS01 strain infection could induce complete autophagy in different cells, we speculate that TMEM39A may play a positive regulatory role in the proliferation of EMCV relying on autophagic signaling. To test this hypothesis, BHK-21 and HEK293 cells were transfected with Entry-TMEM39A or Myc-TMEM39A, respectively. Overexpression of TMEM39A had a significant effect on ATG7 but not ATF4 or ATG3 mRNA expression in BHK-21 cells (Figures 5A−D). In addition, overexpression of TMEM39A upregulated the protein expression levels of LC3B-II and ATG7 and downregulated SQSTM1 in HEK293 cells (Figure 5E). Conversely, TMEM39A knockdown decreased ATG7 and LC3B-II expression and increased SQSTM1 levels (Figure 5F). To assess whether TMEM39A is located downstream of the autophagy signaling pathway, we transfected HEK293 cells with Red-LC3. LC3B overexpression had no effect on TMEM39A mRNA expression (Figures 5G,H). Earlier we showed that TMEM39A could directly interact with EMCV VP1 and VP2 (Figure 1). To verify whether the expression of VP1 or VP2 can affect TMEM39A expression levels, BHK-21 cells were transfected with HA-VP1 or HA-VP2. Interestingly, TMEM39A and ATG7 increased with VP2 overexpression (Figure 6). These findings reveal that overexpression of the EMCV capsid protein, VP2, facilitated the expression of TMEM39A. Overall, these results verify that TMEM39A played a positive regulatory role in the proliferation of EMCV and that TMEM39A can regulate the autophagic machinery.

### Autophagy Blockade Inhibits Encephalomyocarditis Virus Proliferation and Transmembrane Protein 39A Expression

**Figure 7:**
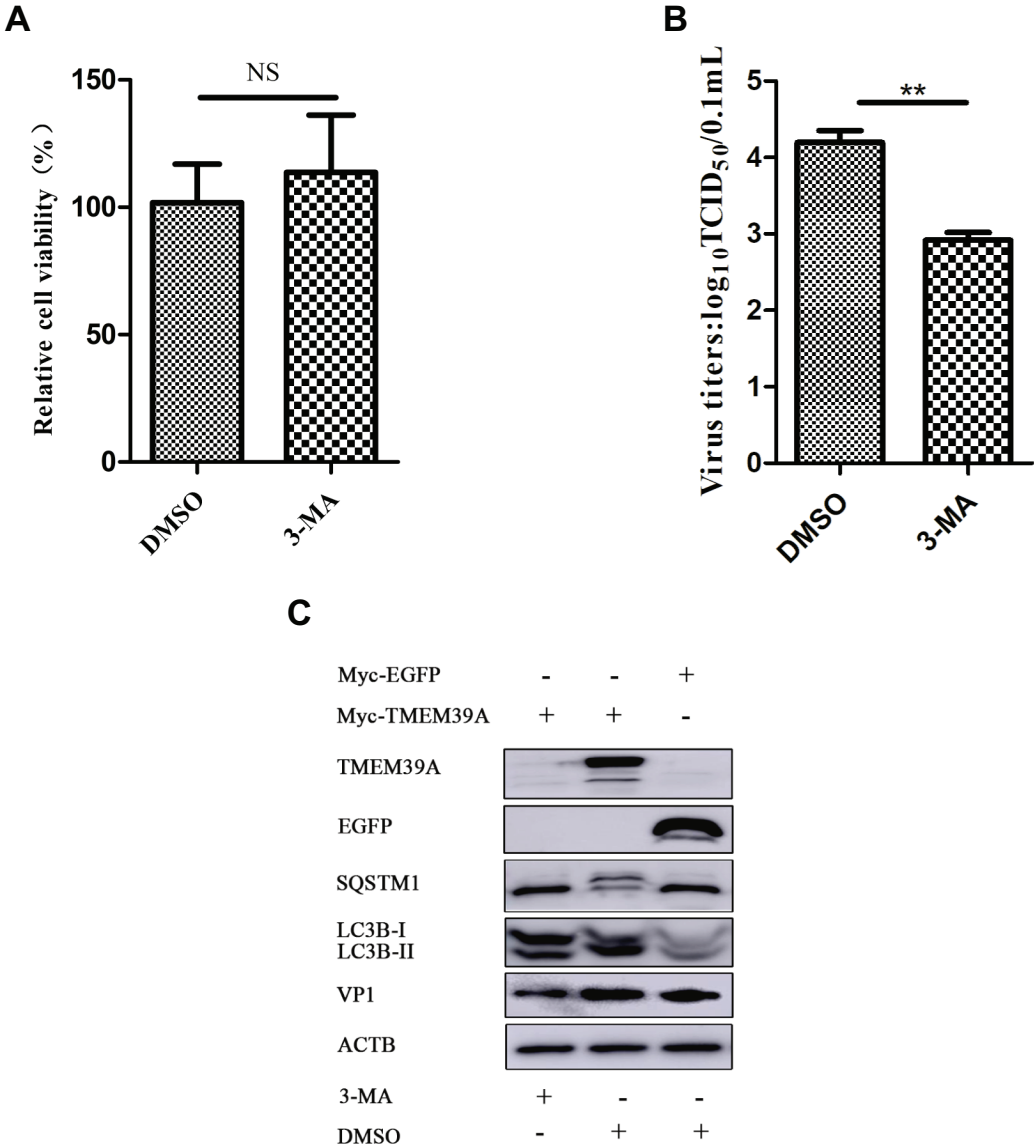
3-MA inhibits the expression of TMEM39A. **(A)** MTT assay of the viability of HEK293 cells treated with 3-MA (5 mM) or DMSO for 6 h. **(B)** TCID_50_ assay for viral titer detection using the Reed-Muench method in the HEK293 cells which were treated with 3-MA (5 mM) or DMSO for 6 h. **(C)** HEK293 cells were first transfected with 2 μg Myc-TMEM39A or Myc-EGFP for 48 h and further infected with 0.0001 MOI EMCV GS01 strain at 24 hpi, and treated 3-MA (5 mM) or DMSO for 6 h. Immunoblotting of VP1, EGFP, LC3B, SQSTM1, and TMEM39A expression in these cells, respectively. ACTB was used as an internal reference. Data are expressed as mean ± SD, n=3. **p < 0.01.

3-Methyladenine (3-MA) is a widely used autophagy inhibitor, which can impede the activity of class III PI3K (Wu et al., 2010). 3-MA alone has no effect on cell viability (Figure 7A). However, 3-MA inhibited the proliferation of EMCV (Figure 7B) and reduced the expression of LC3B-II and VP1 (Figure 7C). Overexpression of TMEM39A upregulated the expression of LC3B-II and downregulated the expression of SQSTM1; however, this phenomenon appeared restitutio ad integrum, and the expression of TMEM39A was inhibited by 3-MA treatment (Figure 7C). These data indicate that the autophagy inhibitor, 3-MA, may inhibit the proliferation of EMCV associated with TMEM39A.

## Discussion

TMEMs are not only used as “carrier terminals” or channels to selectively allow the transport of substances into cells or on biofilms but also as “carriers” to transport substances out of cells (Moon and Fleming, 2011). At present, the function and localization of TMEM family proteins remain unclear. In many cancers, TMEMs can regulate cancer processes in a myriad of ways (Guo et al., 2015; Wrzesiński et al., 2015) and the TMEM39A family member has been a recent attention in cancer research (Tran et al., 2017). TMEM39 family members, TMEM39A and TMEM39B, are formed by alternative splicing (Hao et al., 2015) and belong to the type III-transmembrane protein family. It has been reported to be associated with autoimmune diseases, glioma, and other cancers (Mccauley et al., 2010; Lessard et al., 2012; Varade et al., 2012; Sheng et al., 2015; You et al., 2015; Park et al., 2017; Wagner et al., 2017); however, knowledge regarding the biological function of TMEM39A remains limited. This is the first study to clarify the role of TMEM39A in viral infection and also hints that there may be a interplay between TMEM39A and the ATG7-dependent autophagy pathway. A recent study using large-scale siRNA screening demonstrated that TMEM39A may be involved in Parkin-mediated mitophagy (Orvedahl et al., 2011). However, the underlying molecular mechanism is not yet known. Therefore, it is necessary to investigate the role of TMEM39A on autophagy and mitophagy pathways.

In recent years, studies have shown that many viruses, such as EMCV, hepatitis C virus (HCV), coxsackie virus B (CVB), porcine reproductive and respiratory syndrome virus (PRRSV), foot-and-mouth disease virus (FMDV), and classical swine fever virus (CSFV) can induce autophagy in host cells, while promoting viral replication using autophagy mechanisms (Yoon et al., 2008; Zhang et al., 2011; Gladue et al., 2012; Liu et al., 2012; Mohl et al., 2012; Pei et al., 2014). Subsequently, it has been demonstrated that certain proteins in these viruses play an important role in the induction of autophagy, such as NS4B, a non-structural protein of HCV, and VP2 of FMDV, which are involved in the process of virus-induced autophagy *via* activating the EIF2S1-ATF4 pathway (Li et al., 2009; Sun et al., 2018). One study showed that EMCV HB10 infection can induce autophagy in BHK-21 cells (Zhang et al., 2011). In 2014, our laboratory isolated an EMCV strain from dead piglets in Lanzhou, Gansu province, which was then named the GS01 strain (Feng et al., 2015). The nucleotide homology of the GS01 strain to other strains was 79.9–99.9%, among which VP1 had the most mutations, and this strain also showed high pathogenicity and lethality in mice. Here, we show that the EMCV GS01 strain can also induce complete autophagy in different host cells (BHK-21, C2C12, and HEK293 cells). We found that EMCV infection upregulated the expression of TMEM39A and that overexpression of VP2 facilitated the expression of TMEM39A. Thus, we speculate that the upregulation of TMEM39A expression by EMCV may be related to the role of TMEM39A interaction with VP2; however, the effect of the interaction of VP1 and TMEM39A on EMCV infection is not yet known. Since both EMCV and FMDV are members of the *Picornaviridae* family, and the key amino acids involved in VP2-induced autophagy are the same, we intend to investigate whether the VP2 protein of EMCV can induce autophagy. There have been no reports to date suggesting TMEM39A as an interferon (IFN)-inducible gene. However, GWS studies have implicated TMEM39A in lupus, an autoimmune disease characterized with dysfunctional IFN responses. We have included these references in our manuscript (Lessard et al., 2012; You et al., 2015). Whether IFNs directly plays a role in TMEM39A expression is unknown and would warrant further work. Given that EMCV is commonly used as a model virus to study innate immune responses towards double-stranded RNA (dsRNA) (Carocci and Bakkali, 2012), screening the ability of other RNA viruses and poly I:C, a dsDNA mimic, to regulate TMEM39A expression would be interesting to explore. One of the limitations of our work is the use of already virally transformed cells, such as HEK293 cells. Future work investigating EMCV infection in host primary cells is crucial as this would allow us to pin point the exact transcriptional timing of TMEM39A regulation.

In the present study, we showed that TMEM39A directly interacted with EMCV VP1 and VP2 and played a positive regulatory role in the proliferation of EMCV. This is the first study to clarify the role of TMEM39A in viral infection and provide evidence of a relationship with autophagy pathway. Our findings provide novel ideas for clarifying the role of TMEM39A in the process of viral infection and clinical therapy of certain autoimmune diseases and cancers related to TMEM39A.

## Data Availability Statement

The original data supporting the conclusions of this manuscript will be provided by the authors to any qualified researcher without reservation.

## Author Contributions

XL wrote the draft manuscript. XL and RF designed the experiment. XL, RM, SL, and QL performed experiments and processed the data. HZ, JX, JB, and RF revised and proofread the draft manuscript. AI provided valuable suggestions and comments and was actively involved in the final editing and proofreading of this manuscript. RF supervised the entire process.

### Conflict of Interest

The authors declare that the research was conducted in the absence of any commercial or financial relationships that could be construed as a potential conflict of interest.

## References

[ref1] Bai J. Chen X. H. Jiang K. F. Zeshan B. Jiang P. (2014). Identification of VP1 peptides diagnostic of encephalomyocarditis virus from swine. Virol. J. 11:226. 10.1186/s12985-014-0226-8 25547933 PMC4297377

[ref2] Carocci M. Bakkali K. L. (2012). The encephalomyocarditis virus. Virulence 3, 351–367. 10.4161/viru.20573 22722247 PMC3478238

[ref3] Feng R. F. Wei J. Zhang H. X. Fan J. J. Li X. R. Wang D.. (2015). National serosurvey of encephalomyo-carditis virus in healthy people and pigs in China. Arch. Virol. 160, 2957–2964. 10.1007/s00705-015-2591-z, PMID: 26347283

[ref4] Feng R. F. Zhang H. X. Wei J. Li X. R. Xie J. Y. Li M. S.. (2015). Isolation, molecular and phylogenetic analysis of encephalomyocarditis virus strain GS01 in China. Infect. Genet. Evol. 30, 19–26. 10.1016/j.meegid.2014.12.004, PMID: 25497352

[ref5] Gladue D. P. O’Donnell V. Baker-Branstetter R. Holinka L. G. Pacheco J. M. Fernandez-Sainz I.. (2012). Foot-and-mouth disease virus nonstructural protein 2C interacts with Beclin1, modulating virus replication. J. Virol. 86, 12080–12090. 10.1128/JVI.01610-12, PMID: 22933281 PMC3486479

[ref6] Guo J. Chen L. Luo N. Yang W. Qu X. Cheng Z. (2015). Inhibition of TMEM45A suppresses proliferation, induces cell cycle arrest and reduces cell invasion in human ovarian cancer cells. Oncol. Rep. 33, 3124–3130. 10.3892/or.2015.3902, PMID: 25872785

[ref7] Hao Y. Q. Colak R. Teyra J. Corbi-Verge C. Ignatchenko A. Hahne H.. (2015). Semi-supervised learning predicts approximately one third of the alternative splicing isoforms as functional proteins. Cell Rep. 12, 183–189. 10.1016/j.celrep.2015.06.031, PMID: 26146086

[ref8] Koenen F. (2006). Encephalomyocarditis virus. Boston: Blackwell Science Press.

[ref9] Lessard C. J. Adrianto I. Ice J. A. Wiley G. B. Kelly J. A. Glenn S. B.. (2012). Identification of IRF8, TMEM39A, and IKZF3-ZPBP2 as susceptibility loci for systemic lupus erythematosus in a large-scale multiracial replication study. Am. J. Hum. Genet. 90, 648–660. 10.1016/j.ajhg.2012.02.023, PMID: 22464253 PMC3322228

[ref10] Levine B. (2005). Eating oneself and uninvited guests: autophagy-related pathways in cellular defense. Cell 120, 159–162. 10.1016/j.cell.2005.01.005, PMID: 15680321

[ref11] Levine B. Deretic V. (2007). Unveiling the roles of autophagy in innate and adaptive immunity. Nat. Rev. Immunol. 7, 767–777. 10.1038/nri2161, PMID: 17767194 PMC7097190

[ref13] Li S. S. Ye L. B. Yu X. L. Xu B. Li K. T. Zhu X. D.. (2009). Hepatitis C virus NS4B induces unfolded protein response and endoplasmic reticulum overload response-dependent NF-kappa B activation. Virology 391, 257–264. 10.1016/j.virol.2009.06.039, PMID: 19628242

[ref14] Liu H. M. He X. Y. Song X. F. Xu L. Zhang Y. Zhou G. L. (2016). Isolation and molecular and phylogenetic analyses of encephalomyocarditis virus from wild boar in Central China. Infect. Genet. Evol. 40, 67–72. 10.1016/j.meegid.2016.02.025 26917364

[ref15] Liu Q. H. Qin Y. X. Zhou L. Kou Q. W. Guo X. Ge X. N.. (2012). Autophagy sustains the replication of porcine reproductive and respiratory virus in host cells. Virology 429, 136–147. 10.1016/j.virol.2012.03.022, PMID: 22564420 PMC7111961

[ref16] Luo Y. K. Liang L. Tang Q. H. Zhou L. Shi L. J. Cong Y. Y. (2017). Isolation and characterization of encephalomyocarditis virus from dogs in China. Sci. Rep. 7:438. 10.1038/s41598-017-00435-x 28348405 PMC5428449

[ref17] Mccauley J. L. Zuvich R. L. Beecham A. H. Jager P. L. Konidari I. Whitehead P. L.. (2010). Comprehen-sive follow-up of the first genome-wide association study of multiple sclerosis identifies KIF21B and TMEM39A as susceptibility loci. Hum. Mol. Genet. 19, 953–962. 10.1093/hmg/ddp542, PMID: 20007504 PMC2816610

[ref18] Mohl B. P. Tedbury P. R. Griffin S. Harris M. (2012). Hepatitis C virus-induced autophagy is independent of the unfolden protein response. J. Virol. 86, 10724–10732. 10.1128/JVI.01667-12, PMID: 22837205 PMC3457281

[ref19] Moon C. P. Fleming K. G. (2011). Side-chain hydrophobicity scale derived from transmembrane protein folding into lipid bilayers. Proc. Natl. Acad. Sci. USA 108, 10174–10177. 10.1073/pnas.1103979108 21606332 PMC3121867

[ref20] Oberste M. S. Gotuzzo E. Blair P. Nix W. A. Ksiazek T. G. Comer J. A.. (2009). Human febrile illness caused by encephalomyocarditis virus infection, Peru. Emerg. Infect. Dis. 15, 640–646. 10.3201/eid1504.081428, PMID: 19331761 PMC2671410

[ref21] Orvedahl A. Sumpter R. Jr. Xiao G. H. Ng A. Zou Z. J. Tang Y.. (2011). Image-based genome-wide siRNA screen identifies selective autophagy factors. Nature 480, 113–117. 10.1038/nature10546, PMID: 22020285 PMC3229641

[ref22] Park J. Lee H. Tran Q. Mun K. Kim D. Hong Y.. (2017). Recognition of transmembrane protein 39A as a tumor-specific marker in brain tumor. Toxicol Res. 33, 63–69. 10.5487/TR.2017.33.1.063, PMID: 28133515 PMC5266369

[ref23] Pei J. J. Zhao M. Q. Ye Z. D. Gou H. C. Wang J. Y. Yi L. (2014). Autophagy enhances the replication of classical swine fever virus *in vitro*. Autophagy 10, 93–110. 10.4161/auto.26843 24262968 PMC4389882

[ref24] Schmid D. Münz C. (2007). Innate and adaptive immunity through autophagy. Immunity 27, 11–21. 10.1016/j.immuni.2007.07.004, PMID: 17663981 PMC7118777

[ref25] Sheng Y. J. Xu J. H. Wu Y. G. Zuo X. B. Gao J. P. Lin Y. (2015). Association analyses confirm five susceptibility loci for systemic lupus erythematosus in the Han Chinese population. Arthritis Res. Ther. 17, 85–91. 10.1186/s13075-015-0602-9 25890262 PMC4404072

[ref26] Sun P. Zhang S. M. Qin X. D. Chang X. N. Cui X. R. Li H. T. (2018). Foot-and-mouth disease virus capsid protein VP2 activates the cellular EIF2S1-ATF4 pathway and induces autophagy *via* HSPB1. Autophagy 14, 336–346. 10.1080/15548627.2017.1405187 29166823 PMC5902195

[ref27] Tran Q. Park J. Lee H. Hong Y. Hong S. Park S.. (2017). TMEM39A and human diseases: a brief review. Toxicol Res. 33, 205–209. 10.5487/TR.2017.33.3.205, PMID: 28744351 PMC5523561

[ref28] Varade J. Comabella M. Ortiz M. A. Arroyo R. Fernandez O. Pinto-Medel M. J. (2012). Replication study of 10 genes showing evidence for association with multiple sclerosis: validation of TMEM39A, IL12B and CLBL genes. MultScler 18, 959–965. 10.1177/1352458511432741 22194214

[ref29] Wagner M. Sobczyński M. Bilińska M. Pokryszko-Dragan A. Cyrul M. Kuśnierczyk P.. (2017). Preliminary study on the role of TMEM39A gene in multiple sclerosis. J. Mol. Neurosci. 62, 181–187. 10.1007/s12031-017-0921-1, PMID: 28444502 PMC5486520

[ref30] Wrzesiński T. Szelag M. Cieślikowski W. A. Ida A. Giles R. Zodro E. (2015). Expression of pre-selected TMEMs with predicted ER localization as potential classifiers of ccRCC tumors. BMC Cancer 15:518. 10.1186/s12885-015-1530-4 26169495 PMC5015219

[ref31] Wu Y. T. Tan H. L. Shui G. Bauvy C. Huang Q. Wenk M. R.. (2010). Dual role of 3-methyladenine in modulation of autophagy *via* different temporal patterns of inhibition on class I and III phosphor-inositide 3-kinase. J. Biol. Chem. 285, 10850–10861. 10.1074/jbc.M109.080796, PMID: 20123989 PMC2856291

[ref32] Xiao Y. Z. Liu H. Yu J. J. Zhao Z. L. Xiao F. Xia T. T. (2016). MAPK1/3 regulate hepatic lipid metabolism *via* ATG7-dependent autophagy. Autophagy 12, 592–593. 10.1080/15548627.2015.1135282 26760678 PMC4836014

[ref33] Yoon S. Y. Ha Y. E. Choi J. E. Ahn J. Lee H. Kweon H. S.. (2008). Coxsackievirus B4 uses autophagy for replication after calpain activation in rat primary neurons. J. Virol. 82, 11976–11978. 10.1128/JVI.01028-08, PMID: 18799585 PMC2583648

[ref34] You Z. Y. Xu Y. F. W W. Zhou L. Li J. Zhou T. H. (2019). TP53INP2 contributes to autophagosome formation by promoting LC3-ATG7 interaction. Autophagy 15, 1309–1321. 10.1080/15548627.2019.1580510 30767704 PMC6613902

[ref35] You Y. Zhai Z. F. Chen F. R. Chen W. Hao F. (2015). Autoimmune risk loci of IL12RB2, IKZF1, XKR6, TMEM39A and CSK in Chinese patients with systemic lupus erythematosus. Tissue Antigens 85, 200–203. 10.1111/tan.12522 25720506

[ref36] Zhang Y. N. Li Z. C. Ge X. N. Guo X. Yang H. C. (2011). Autophagy promotes the replication of encephalomyocarditis virus in host cells. Autophagy 7, 613–628. 10.4161/auto.7.6.15267 21460631

[ref37] Zhang L. Qi Y. Luo L. Sun J. G. Yuan W. Z. (2017). Development and application of an indirect ELISA for the detection of antibodies against encephalomyocarditis virus. Biomed Rep. 7, 423–428. 10.3892/br.2017.989, PMID: 29109860 PMC5663986

